# Concordance of Abundance for Mutational *EGFR* and Co-Mutational *TP53* with Efficacy of *EGFR*-TKI Treatment in Metastatic Patients with Non-Small-Cell Lung Cancer

**DOI:** 10.3390/curroncol30090616

**Published:** 2023-09-15

**Authors:** Youping Wang, Hong Liu, Ningjuan Yu, Xueping Xiang

**Affiliations:** 1 Department of Medical Oncology, The Second Affiliated Hospital, School of Medicine, Zhejiang University, Hangzhou 310009, China; 2cynthia@163.com; 2 State Key Laboratory of Oncology in South China, Collaborative Innovation Center for Cancer Medicine, Department of Medical Oncology, Sun Yat-sen University Cancer Center, Guangzhou 510060, China; liuhong2@sysucc.org.cn; 3 Department of Pathology, The Second Affiliated Hospital, School of Medicine, Zhejiang University, Hangzhou 310009, China; xiangxueping@zju.edu.cn

**Keywords:** non-small-cell lung adenocarcinoma, *EGFR* mutation abundance, *EGFR*-TKIs, *TP53* co-mutation

## Abstract

The present study aimed to investigate the influence of the mutation abundance of the epidermal growth factor receptor (*EGFR*) and its co-mutation with *TP53* on the therapeutic efficacy of tyrosine kinase inhibitor (TKI) treatment in patients with metastatic lung adenocarcinoma (LUAD). In total, 130 patients (January 2018-September 2022) with metastatic LUAD from the Second Affiliated Hospital of Zhejiang University were included. Kaplan–Meier analysis was performed to measure the duration of drug application (DDA) and the log-rank test was used to compare differences. Univariate and multivariate analyses of Cox proportional hazard regression models were used to evaluate the association between the relevant clinicopathological factors and DDA. Hazard ratios with 95% confidence intervals were also calculated. Among the 130 patients who were treated with first-generation *EGFR*-TKIs, 86 showed high-*EGFR* mutation abundance (>22.0%) and 44 showed low-*EGFR* mutation abundance (≤22.0%). Patients in the high-*EGFR* group had a greater DDA than those in the low-*EGFR* group (*p* < 0.05). The results of the subgroup analysis were consistent with those of the total mutation population (exon19: >18.5% vs. ≤18.5%, 14 months vs. 10 months, *p* = 0.049; exon21: >22.0% vs. ≤22.0%, 15 months vs. 9 months, *p* = 0.005). In addition, the mutation abundance of *TP53* was negatively correlated with the DDA (*p* < 0.05). Patients in the combination group had a better DDA than those in the monotherapy group (*p* < 0.05). Subgroup analysis showed that, among the low mutation abundance of the *EGFR* exon 21 or 19 cohort, the combination group had a better DDA than the monotherapy group (*p* < 0.05). An *EGFR* mutation abundance greater than 22.0% was a positive predictor of DDA in patients with metastatic LUAD. However, a *TP53* mutation abundance higher than 32.5% could reverse this situation. Finally, first-line treatment with *EGFR*-TKIs plus chemotherapy is a potential treatment strategy for patients with low-abundance *EGFR* mutations.

## 1. Introduction

Lung cancer is a common and fatal malignant tumor found worldwide [1]. Non-small-cell lung cancer (NSCLC) is the most common histological type of lung cancer, accounting for more than 85% of lung cancer cases [2]. Epidermal growth factor receptor (*EGFR*) is a common driver gene in NSCLC, and approximately 20% of NSCLC patients have activating mutations in *EGFR*, particularly those who have an adenocarcinoma histology and have never smoked or have a history of light smoking [3]. *EGFR*-mutated NSCLC is a genetically heterogeneous disease with more than 200 distinct mutations. Deletion of exon 19 and L858R point mutation in exon 21 are dominant [4,5]. In the field of NSCLC, progress in genetic testing technology and the discovery of new tumor driver genes have promoted the development and clinical application of NSCLC-targeted therapeutic drugs. *EGFR* tyrosine kinase inhibitors (TKIs) can remarkably improve progression-free survival (PFS) and are recommended as the first-line treatment for *EGFR*-mutated metastatic NSCLC according to the NCCN guidelines [6,7]. To date, several TKIs have been approved for the treatment of *EGFR*-mutated NSCLC, including first-generation (Erlotinib, Gefitinib), second-generation (Afatinib), and third-generation (Osimertinib) TKIs [8,9]. Each of these TKIs works by targeting specific mutations in the EGFR gene, inhibiting the tyrosine kinase activity, and blocking downstream signaling pathways that promote cancer growth. First-generation TKIs have shown efficacy in the initial treatment of EGFR-mutated NSCLC, while second-generation TKIs offer broader coverage against EGFR mutations. Osimertinib, a third-generation TKI, specifically targets the T790M resistance mutation, which is common in patients who develop acquired resistance to first-generation TKIs. It has demonstrated remarkable effectiveness in both first- and second-line treatment settings.

However, there are significant differences in the clinical efficacy of TKIs among individuals. Few studies have been conducted on the primary drug resistance of *EGFR*-TKIs, and the related mechanisms are unclear. Previous studies have shown that the efficacy of *EGFR*-TKIs is highly heterogeneous [10]. Co-mutations play an important role in the response and resistance to *EGFR*-TKIs in *EGFR*-mutant NSCLC, partially explaining the effect of heterogeneity [11,12]. The majority of metastatic *EGFR*-mutant lung adenocarcinomas (LUAD) harbor one or more co-mutations, and *TP53* is a common co-mutated gene in *EGFR*-mutant NSCLC, accounting for 55–65% of cases [13,14]. Tumors bearing co-mutations in *TP53* exhibit high genomic instability and somatic mutation burden [15]. Multiple clinical studies have identified *TP53* co-alteration as a negative prognostic marker in LUAD with *EGFR* mutations and as a predictor of poor clinical outcomes of *EGFR*-TKI treatment [16].

However, intratumoral heterogeneity is another factor limiting the efficacy of *EGFR*-TKIs [17]. Several reports have focused on the quantitative assessment of genetic mutations instead of qualitative tests [18]. The abundance of *EGFR* mutations differs among tumors, or in different samples obtained from the same tumor, because mutant and wild-type (WT) *EGFR* can exist concurrently in the same primary NSCLC [17]. Wu et al. first proposed individual differences in the abundance of *EGFR* mutations in NSCLC by direct sequencing and using the amplification refractory mutation system (ARMS), which indicated that patients with high mutation abundance treated with *EGFR*-TKIs had significantly longer PFS than those with low mutation abundance [18]. However, determining the mutation abundance in tumor tissues to investigate the difference in sensitivity between ARMS and PCR is difficult. The application of ARMS is primarily limited by false positive results caused by non-specific primers that may be produced by WT DNA, as well as the missed detection of low-frequency mutations [19,20]. Lung cancer cells have a high incidence of *EGFR* amplification, which can affect the abundance of *EGFR* mutations, as determined by the ARMS assay. Therefore, a quantitative and highly sensitive method is necessary to detect the abundance of *EGFR* mutants.

Compared to traditional genetic testing methods, second-generation gene sequencing (NGS) has several advantages. It can simultaneously detect point mutations, insertion/deletion, copy number variation, and gene rearrangement of four variant forms, from several genes to hundreds of genes in whole exomes or whole genomes, and it can also provide comprehensive gene mutation data [21]. In patients previously diagnosed with metastatic NSCLC who were found to be negative for *EGFR* and anaplastic lymphoma kinase (ALK) variants using traditional single-gene testing, 17.4% carried at least one *EGFR* or ALK variant, as outlined by NGS [22]. Investigators performed NGS analysis in patients with LUAD who showed negative genetic variation (*EGFR*/*KRAS*) using traditional genetic testing methods, and 31–65% of the samples showed genetic variation; some patients received approved drugs in accordance with the NCCN guidelines and achieved clinical benefit [23,24]. Therefore, in the case of false negative results from a non-NGS technology, NGS retesting is recommended for patients with negative traditional testing methods [25].

Currently, no reports have been found on whether the co-mutation abundance of *EGFR* and *TP53* affects the efficacy of *EGFR*-TKIs. Thus, this study aimed to identify the role of *EGFR* mutation abundance using NGS in the prognosis of metastatic LUAD and the landscape and influence of its co-mutations, as well as to explore therapeutic modalities for low-*EGFR* mutation abundance. The detection of mutation abundance is of great significance for individualized treatment of patients. Therefore, the population carrying *EGFR* mutations can be subdivided according to the mutation abundance, thereby accurately distinguishing the targeted therapy population.

## 2. Materials and Methods

### 2.1. Patients

Data on metastatic LUAD patients with *EGFR* mutations were retrospectively extracted from the Second Affiliated Hospital of Zhejiang University School of Medicine (SAHZU) between January 2018 and September 2022. Detailed inclusion criteria were as follows: (a) patients with stage IV LUAD; (b) histopathological examination showing lung adenocarcinoma; (c) all tissue samples for NGS detection evaluated with HE staining by well-trained, experienced pathologists to ensure that the tumor cell content was greater than 50%; (d) NGS analysis showing *EGFR* exon 19/21 mutation, with conclusive mutation abundance data; (e) oral *EGFR*-TKI therapy used as first-line treatment for more than 1 month; (f) patients who underwent chest and abdominal CT, brain MRI, and other imaging examinations every 2–3 months to evaluate the potential therapeutic effects on target or non-target lesions during *EGFR*-TKI administration; and (g) complete clinical information. The exclusion criteria were as follows: (a) NGS test after treatment (*n* = 4); (b) second- or third-generation *EGFR*-TKIs as first-line treatment (*n* = 30); (c) non-*EGFR* exon19/21 mutation (*n* = 5); (d) non-tissue samples for NGS (*n* = 41); and (e) *EGFR* T790 mutation (*n* = 5) (Supplementary Figure S1). Finally, 130 metastatic LUAD patients with *EGFR* mutations who were treated with *EGFR*-TKIs as the first-line treatment were included. Of these, 55 had *TP53* mutations. To determine the cut-off values, the maximum mutation abundance was incorporated into the statistical analysis for patients with more than one mutation type. The study protocol was approved by the Ethical Committee of SAHZU. 

### 2.2. Abundance of EGFR Mutations

Common *EGFR* mutations, including those in exons 19 and 21, were detected using NGS. NGS was performed using the TruSight One Sequencing Panel Kit (Illumina, San Diego, CA, USA). Briefly, DNA was extracted from tissue samples using a DNA tissue extraction reagent kit. The ILLUMINA NOVASEQ 6000 sequencing platform was used for the enrichment of targeted region hybridization and high-throughput parallel sequencing of disease-related gene mutations. The average sequencing depth was 2000×, with a sensitivity of 0.1%. A total of 641 disease-related gene mutations were identified. The pathogenicity of mutations was assessed based on international databases (ClinVar, Catalog of Somatic Mutations in Cancer (COSMIC), and OncoKB) and then filtered through the company’s self-built database and methods. Using X-tile software (version 3.6.1), the optimal cut-off values of *EGFR* exon 19 and exon 21 mutation abundance for the duration of drug application (DDA) were 18.5% and 22.0%, respectively. Moreover, the optimal cutoff values of *EGFR* and *TP53* mutation abundance for DDA were 22.0% and 32.5%, respectively.

### 2.3. Statistical Analysis

Categorical variables are reported as numbers and percentages, and continuous variables are reported as means and standard deviations. DDA was defined as the treatment time from the initial use of one *EGFR*-TKI to the cessation of the *EGFR*-TKI or the change to another *EGFR*-TKI until disease progression or death from any cause. The DDA times were analyzed using Kaplan–Meier analysis and compared among the groups using the log-rank test. Propensity score matching (PSM) analysis was performed to reduce the impact of other clinical characteristics on DDA. PSM analysis is a statistical technique used to reduce bias and confounding in observational studies. It aims to match individuals or groups based on their propensity scores, which estimate the probability of receiving a particular treatment or exposure. The propensity score is calculated using observed characteristics or covariates that are believed to influence both the treatment assignment and outcome. PSM analysis helps balance the distribution of confounding factors across treatment groups, making it a valuable method for drawing causal inferences from observational data. Univariate and multivariate analyses of Cox proportional hazard regression models were used to evaluate the association between the relevant clinicopathological factors and DDA. Hazard ratios (HRs) with 95% confidence intervals (CIs) were calculated. All statistical analyses were performed using R software (version 4.2.1). Statistical significance was defined as a two-sided *p* value < 0.05.

## 3. Results

### 3.1. Characteristics of the Included Patients

Among 130 patients with metastatic LUAD who were treated with first-generation *EGFR*-TKIs as a first-line treatment, 86 showed high-*EGFR* mutation abundance (high-*EGFR* group, >22.0%), and 44 showed low-*EGFR* mutation abundance (low-*EGFR* group, ≤22.0%). Patient characteristics are shown in Table 1. Icotinib was the preferred first-generation TKI for first-line treatment, accounting for 70% of the high-*EGFR* group and 61% of the low-*EGFR* group, followed by gefitinib and erlotinib. Most patients were treated with *EGFR*-TKI monotherapy, regardless of the *EGFR* mutation abundance, particularly in the high-*EGFR* group (88%). Of the 130 patients, 43 received third-generation *EGFR*-TKIs as a second-line treatment following first-line first-generation *EGFR*-TKIs. Most patients achieved a complete/partial response after first-line treatment, accounting for 77% of the high-*EGFR* group and 77% of the low-*EGFR* group. More than 80% of patients had one *EGFR* mutation. Exons 19 and 21 were the most common mutation types of *EGFR*, and the number of patients with only exon 19 mutations and exon 21 mutations was 55 and 63, respectively; 12 of these cases were exon 19/21 combined with other non-T790 mutations. More than half of the patients (51%) had a combined *TP53* mutation in the high-*EGFR* group.

**Figure 1:**
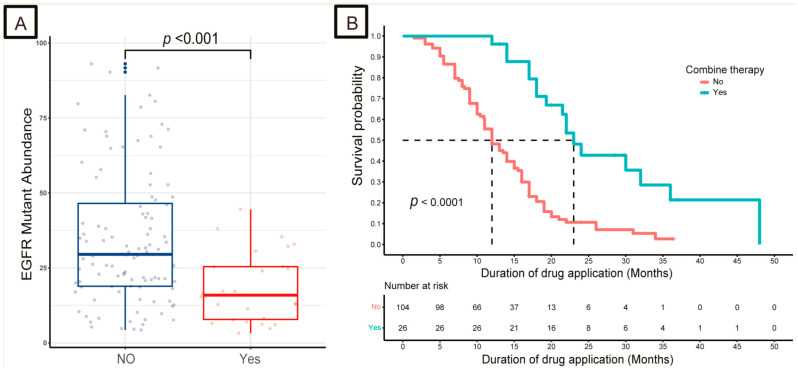
Distribution of *EGFR* mutation abundance and Kaplan–Meier survival curves for duration of drug application (DDA). (**A**) The distribution of *EGFR* mutation abundance of LUAD patients in the single group and the combination group. (**B**) Kaplan–Meier survival curves for duration of drug application (DDA) in the single group and the combination group. *EGFR*, epidermal growth factor receptor.

A total of 26 patients underwent combination therapy (*EGFR*-TKIs plus chemotherapy or anti-angiogenesis (the combination group)) and 104 patients underwent *EGFR*-TKI monotherapy (the single group) as first-line treatment. The abundance of *EGFR* mutations was lower in the combination group than in the single group (*p* < 0.05, Figure 1A), and patients in the combination group had better DDA than those in the single group (23 months vs. 12 months, *p* < 0.05, Figure 1B). No difference in *EGFR* mutation abundance or DDA was observed between *EGFR* mutation numbers and types (Supplementary Figure S2).

### 3.2. Impact of EGFR and TP53 Mutation Abundance on Response to TKIs

Based on X-tile software, the optimal cut-off values of *EGFR* exon 19 and exon 21 mutation abundance for DDA were 18.5% and 22.0%, respectively.

**Figure 2:**
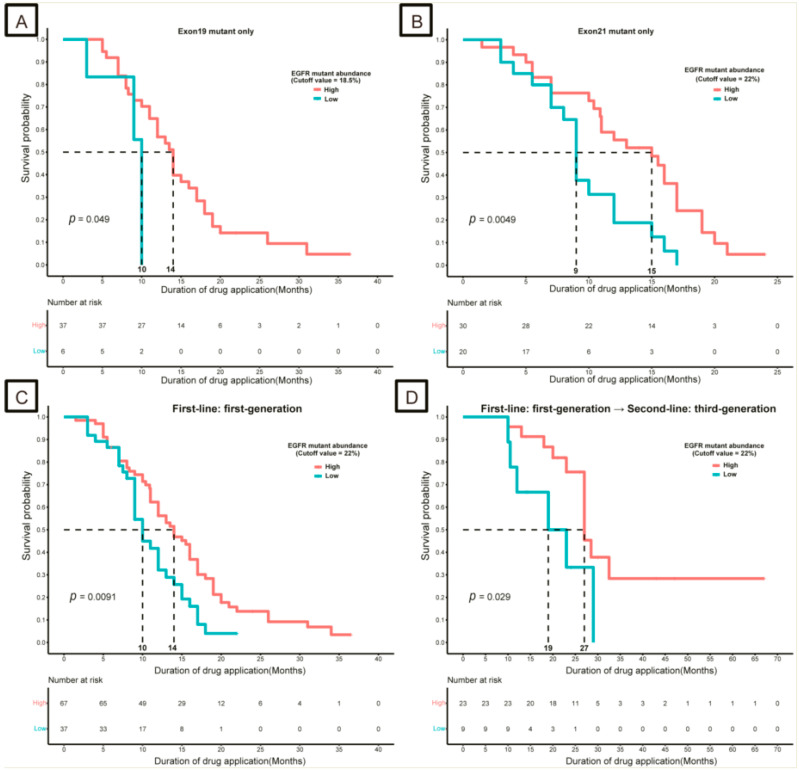
Kaplan–Meier survival curves for the duration of drug application (DDA) in different *EGFR* mutation abundance groups. (**A**) Only *EGFR* exon 19 mutation cohort; (**B**) only *EGFR* exon 21 mutation cohort; (**C**) first-generation *EGFR*-TKIs as first-line treatment cohort; (**D**) first-generation *EGFR*-TKIs for first-line treatment, followed by third-generation *EGFR*-TKIs for second-line treatment cohort. *EGFR*, epidermal growth factor receptor.

Patients with high-*EGFR* exon 19 mutations (>18.5%) had a greater DDA than those with low abundance (≤18.5%) (14 months vs. 10 months, *p* = 0.049; Figure 2A). Consistently, high -*EGFR* exon 21 mutation abundance (>22.0%) was also associated with good DDA (15 months vs. 9 months, *p* = 0.005; Figure 2B).

Based on X-tile software, the optimal cutoff values of *EGFR* and *TP53* mutation abundance for DDA were 22.0% and 32.5%, respectively.

A total of 104 patients who received first-generation TKIs as first-line treatment provided tissue samples for NGS, and high-*EGFR* mutation abundance (>22.0%) was associated with good DDA (14 months vs. 10 months, *p* = 0.009; Figure 2C). In addition, 32 patients received first-generation *EGFR*-TKIs as the first-line treatment, followed by third-generation *EGFR*-TKIs as the second-line treatment. Similarly, in these patients, a high abundance (>22.0%) of *EGFR* mutations was associated with good DDA, compared with a low abundance (≤22.0%; 27 months vs. 19 months, *p* = 0.029; Figure 2D).

**Figure 3:**
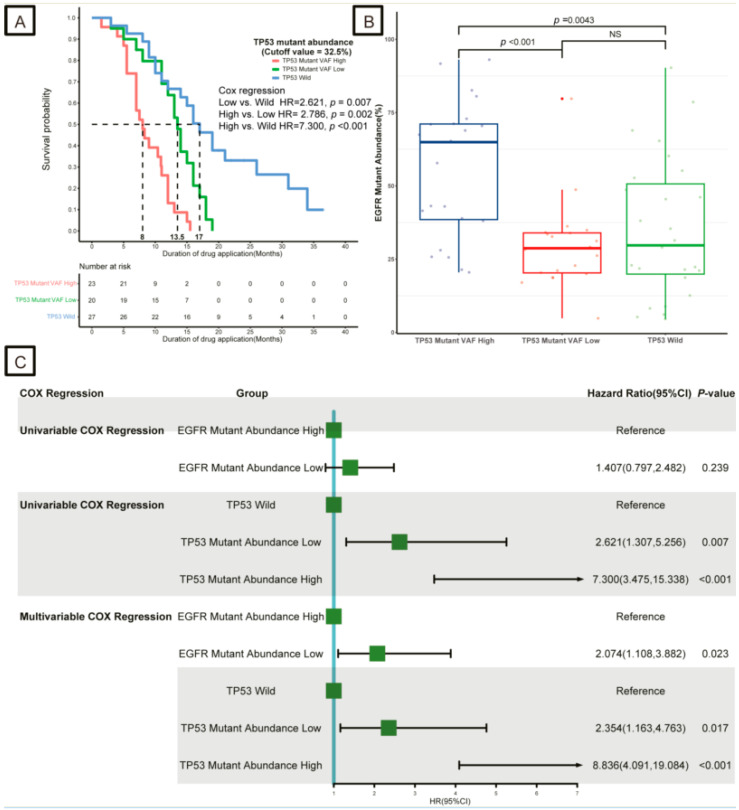
Survival analysis results in different *EGFR* and *TP53* mutation abundance groups. (**A**) Kaplan–Meier survival curves for the duration of drug application (DDA) of different *TP53* mutation abundance groups in the first-generation *EGFR*-TKIs as first-line treatment cohort. (**B**) The distribution of *EGFR* mutation abundance of different *TP53* mutation abundance groups in the first-generation *EGFR*-TKIs as first-line treatment cohort. (**C**) Forest plot for univariable and multivariable Cox regression analysis results. *EGFR*, epidermal growth factor receptor; NS, not significant; HR, hazard ratio; CI, confidence interval.

Among these patients, 23 had a high mutation abundance of *TP53* (>32.5%, the high-*TP53* group), 20 had a low mutation abundance of *TP53* (≤32.5%, the low-*TP53* group), and 27 had no *TP53* mutation (WT group). The high-*TP53* group had worse DDA (8 months) than those in the low-*TP53* group (13.5 months, Cox regression analysis, *p* < 0.05) and WT group (17 months, Cox regression analysis, *p* < 0.05; Figure 3A). In addition, patients in the high-*TP53* group had a higher *EGFR* mutation abundance of *EGFR* compared with those in the low-*TP53* group and WT groups (*p* < 0.05, Figure 3B).

PSM was performed to reduce the impact of other clinical characteristics on the DDA. After PSM analysis, 39 patients were included in the high-*EGFR* and low-*EGFR* groups and 14 patients were included in the high-*TP53* group and low-*TP53* groups, respectively. The characteristics of the two groups were matched (*p* > 0.05) and are presented in Supplementary Tables S1 and S2. Similar results showed that the high mutation abundance of *EGFR* (>22.0%) was associated with a greater DDA (17 months vs. 12 months, *p* = 0.011), and a high mutation abundance of *TP53* (>32.5%) was associated with poor DDA (13.5 months vs. 8.3 months, *p* = 0.004) (Supplementary Figure S3).

To explore the association between *EGFR* and *TP53* mutations, univariate and multivariate Cox regression analyses were performed in the tissue sample cohort. Univariate Cox regression analysis showed that the *EGFR* mutation abundance (low vs. high, HR [95% CI], 1.407 [0.797, 2.482], *p* > 0.05) was not an independent predictor of DDA compared to *TP53* mutation abundance (low vs. WT, HR [95% CI], 2.621 [1.307, 5.256], *p* = 0.007; high vs. WT, HR [95% CI], 7.300 [3.475, 15.338], *p* < 0.001). However, based on multivariate Cox regression analysis, *EGFR* mutation abundance (low vs. high, HR [95% CI], 2.074 [1.108, 3.882], *p* = 0.023) and *TP53* mutation abundance (low vs. WT, HR [95% CI], 2.354 [1.163, 4.763], *p* = 0.017; high vs. WT, HR [95% CI], 8.836 [4.091, 19.084], *p* < 0.001) were independent predictors of DDA (Figure 3C).

### 3.3. Strategies for Patients with Low-Abundance Mutations

**Figure 4:**
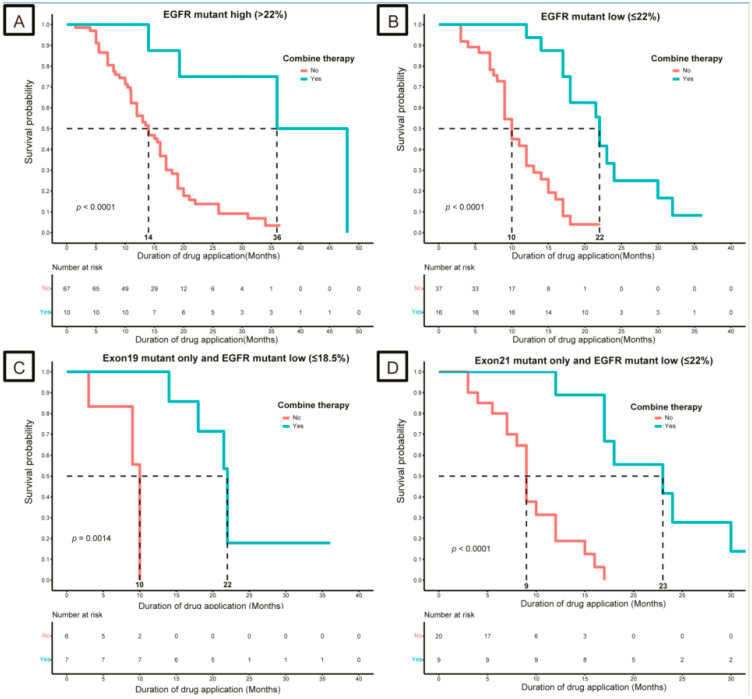
Kaplan–Meier survival curves for duration of drug application (DDA) based on whether or not combination therapy was received. (**A**) High-*EGFR* mutation abundance (>22.0%) cohort; (**B**) low-*EGFR* mutation abundance (≤22.0%) cohort; (**C**) only *EGFR* exon 19 mutation and low-*EGFR* mutation abundance (≤18.5%) cohort; (**D**) only *EGFR* exon 21 mutation and low-*EGFR* mutation abundance (≤22.0%) cohort. *EGFR*, epidermal growth factor receptor.

Patients in the combination group were associated with good DDA compared with those in the single group, regardless of whether they were high or low (*p* < 0.05, Figure 4A,B). Among the *EGFR* exon 19 mutations and low-*EGFR* mutation abundance (≤18.5%) cohort, the combination group had a better DDA than the monotherapy group in tissue samples (22 months vs. 10 months, *p* < 0.05, Figure 4C). Similar results were observed only in the *EGFR* exon 21 mutation and low-*EGFR* mutation abundance (≤22.0%) cohorts (*p* < 0.05, Figure 4D).

## 4. Discussion

For metastatic NSCLC with *EGFR* mutations, the first-line use of TKI greatly improves the survival time and quality of life of patients [26,27]. However, some patients have a poor response to TKIs, and approximately 20% of patients develop primary resistance to *EGFR*-TKIs [6], indicating unknown mechanisms restricting the efficacy of TKIs. Intratumoral heterogeneity is an important cause of treatment failure and refers to the presence of heterogeneous targeted molecules (such as *EGFR*) in tumor tissues; that is, *EGFR*-mutant and WT clones in the same tumor [28,29]. Therefore, different levels of mutant molecules may be part of the variable response to TKIs. In this study, the *EGFR* mutation abundance was different in different samples from the same patient and at different sites in the same sample. Previous studies have demonstrated that the abundance of *EGFR*-activating mutations is significantly associated with an objective response to *EGFR*-TKIs, and the PFS of patients with high mutation abundance is significantly better than that of patients with low mutation abundance [30,31]. In addition, the emergence of the T790M mutation in the kinase domain of EGFR is another common and known mechanism of acquired resistance, occurring in approximately 60% of tumors resistant to first-generation EGFR-TKIs [32]. Therefore, in this study, we excluded patients with the EGFR T790M mutation.

However, factors affecting the mutation abundance and efficacy of *EGFR*-TKIs have not been explored in previous studies. Therefore, in this study, we aimed to investigate the influence of sensitive mutation abundance and *EGFR* co-mutations on the therapeutic efficacy of TKIs. Consistent with previous reports, we found that a high *EGFR*-mutation abundance determined by NGS was positively associated with the efficacy of *EGFR*-TKIs. The cut-off values calculated by ROC analysis were used to divide *EGFR*-mutant NSCLC into high (≥22.0%) and low (<22.0%) groups to minimize the number of false positives. Subgroup analysis showed that the cutoff values for the *EGFR* exon 19 and 21 mutation cohorts were 18.5% and 22.0%, respectively. Patients in the high-*EGFR* mutation group had greater DDA than those in the low-*EGFR* mutation group. Further analysis showed that high-*EGFR* mutation abundance was also positively associated with DDA in the *EGFR* exon 21 and exon 19 mutation cohorts. In contrast to Zhou’s reports [18], no difference in *EGFR* mutation abundance was observed between the different *EGFR* mutation types (exons 19 and 21). This may be due to the small number of patients in the cohort.

In *EGFR*-mutant lung cancers, several reports have suggested that concurrent *TP53* alterations are associated with a lower likelihood of response to *EGFR*-TKIs and shorter overall survival [33,34]. Several studies have shown that *TP53* mutations are important factors in primary *EGFR*-TKI resistance [35,36]. We analyzed the effect of *TP53* mutations on the response to first-line TKIs in patients with *EGFR*-mutated LUAD. The results showed that *TP53* mutations reduced the TKI responsiveness. Patients in the high-*TP53* group (>32.5%) had a worse DDA (8 months) than those in the low-*TP53* group (≤32.5%; 13.5 months) and WT group (17 months). A statistically significant difference was observed between low *TP53* mutation abundance and WT *TP53* on DDA (*p* = 0.002). PSM analysis also verified these results. Using univariate Cox regression analyses, we also explored the association between *EGFR* and *TP53* in the *TP53* mutation cohort and confirmed that *TP53* mutation abundance was an independent predictor of DDA. The abundance of *EGFR* and *TP53* mutations was associated with DDA, according to multivariable Cox regression analyses. In addition, patients in the high-*EGFR* group had poor DDA, which was accompanied by high *TP53* mutation abundance, indicating that high *TP53* abundance was associated with poor prognosis. Consistent with our research, Xu et al. [37] reported that patients with a short-term response to *EGFR*-TKIs (PFS < 6 months) had a high incidence of *TP53* co-mutation using NGS (88% vs. 13%, *p* < 0.001) in metastatic NSCLC patients with *EGFR* exon 21 and exon 19 mutations, indicating that *TP53* co-mutation is a poor prognostic factor in *EGFR*-mutant NSCLC. However, *TP53* abundance was not analyzed in the present study. Multiple studies have shown that *TP53* mutations can be used as a poor prognostic factor in metastatic NSCLC, and mutations in exons at different sites have different effects on clinical prognosis in clinical studies with small samples [38,39,40,41]. Therefore, refined typing of *EGFR*-*TP53* co-mutations has important clinical value for the future clinical prognosis of NSCLC diagnosis and treatment. This method will expand the sample size and promote the analysis of the influence of *TP53* mutation types on the prognosis and efficacy of *EGFR*-TKIs. Apart from *EGFR* and *TP53*, other mutation types are also of great importance, but the number is small and does not constitute a statistical sample. Therefore, further research analyzing the relationship between *EGFR* and other mutation types with a large cohort should be carried out.

Based on the above-mentioned analysis, primary resistance to *EGFR*-TKIs and poor prognosis in patients with *EGFR*-sensitive mutations were partly due to the low abundance of *EGFR* mutations and co-mutations of *TP53*. Therefore, patients must develop a comprehensive *EGFR*-TKI-based treatment program. The NEJ009 study [42] compared first-line treatment with gefitinib with or without chemotherapy for metastatic NSCLC with *EGFR* mutations, and the results showed that the combination group had a higher ORR and better PFS than the gefitinib group (*p* < 0.001). Furthermore, Yan et al. [43] showed that first-line treatment with *EGFR*-TKI plus chemotherapy significantly improved PFS and OS because of the low abundance of *EGFR* mutations; however, the low abundance of the cutoff value (<10%) was defined based on the ARMS detection technique. We also conducted an exploratory analysis of the treatment strategies for patients with low-abundance mutations. We found that the patients in the combination group (chemotherapy or anti-angiogenesis) had better DDA than those in the single-TKI group, regardless of the abundance of *EGFR* mutations, and the statistical difference in DDA was more significant in the low-abundance *EGFR* (<22.0%) group (*p* < 0.0001, Figure 4A,B). Subgroup analysis showed that, for patients with a low abundance of *EGFR* exon 19 mutations (≤18.5%), the combination group had a better DDA than the monotherapy group in all samples (*p* < 0.05, Figure 4C). In addition, the same results were observed in the low-abundance *EGFR* exon 21 cohort (≤22.0%, *p* < 0.05, Figure 4D). Combination therapy is promising, and positive results are expected to confirm the superiority of this regimen. The OPAL study [44], a phase 2 study of osimertinib in combination with platinum and pemetrexed in patients with previously untreated *EGFR*-mutated advanced non-squamous non-small-cell lung cancer, consistently showed excellent efficacy, with the ORR, CRR, and DCR being 90.9% (95% confidence interval [CI], 84.0–97.8), 3.0% (0.0–7.2), and 97.0% (92.8–100.0), respectively. Our study provides a therapeutic approach for patients with a low abundance of *EGFR*-sensitive mutations. However, the study data were limited to DDA and further clinical studies must be conducted to confirm whether combination therapy has a survival benefit.

Detection of mutation abundance is of great importance for the individualized treatment of patients. Therefore, in a population carrying *EGFR*-sensitive mutations, patients can be further subdivided based on their mutation abundance to accurately determine the treatment population and timing. We selected DDA as the primary endpoint because it is more accurate and consistent with clinical practice than PFS. In addition, the efficacy and toxicity, as well as some cases of slow progression, must be considered. However, the relationship between *TP53* abundance and different *EGFR* mutation sites, as well as the different sites of *TP53* mutations and DDA, should be further confirmed by expanding the sample size. Additionally, due to the sample size limitation, we only focused on analyzing the cohort using first-generation TKIs and first-generation TKIs, followed by third-generation TKIs.

## 5. Conclusions

*EGFR*-TKIs are the current standard first-line treatment regimen for metastatic *EGFR* mutations in NSCLC. Primary drug resistance caused by *EGFR*/*TP53* co-mutation is a significant limitation in molecular targeted therapy, particularly for high-abundance *TP53* mutations. In this study, we found that an *EGFR* mutation abundance greater than 22.0% was a positive predictor of DDA in patients with metastatic LUAD. However, a *TP53* mutation abundance higher than 32.5% could reverse this situation. Combination therapy (chemotherapy or anti-angiogenesis) may be an effective treatment strategy for patients with low-abundance *EGFR* mutations or high-abundance *TP53* mutations. However, large, prospective, randomized, controlled clinical studies must be conducted to validate the clinical implications of our results.

## Figures and Tables

**Table 1:** Characteristics of the included patients.

Characteristics	High-*EGFR* Group (N = 86)	Low-*EGFR* Group (N = 44)
Age (years, range)	63 (56, 69)	67 (56, 73)
Gender		
Male	39 (45%)	18 (41%)
Female	47 (55%)	26 (59%)
First-line therapy		
Icotinib	60 (70%)	27 (61%)
Gefitinib	26 (30%)	16 (36%)
Erlotinib	0 (0%)	1 (2.3%)
Combination therapy (first-line)		
No	76 (88%)	28 (64%)
Chemotherapy	4 (4.7%)	8 (18%)
Target therapy	5 (5.8%)	7 (16%)
Chemotherapy plus target therapy	1 (1.2%)	1 (2.3%)
Second-line therapy		
Third-generation TKIs	30 (35%)	13 (30%)
Osimertinib	27 (31%)	10 (23%)
Almonertinib	2 (2.3%)	1 (2.3%)
Furmonertinib	1 (1.2%)	2 (4.5%)
Other or unknown	56 (65%)	31 (70%)
Best therapy response (first-line)		
CR/PR	66 (77%)	34 (77%)
SD	19 (22%)	7 (16%)
PD	1 (1.2%)	3 (6.8%)
*EGFR* mutant number		
1	74 (86%)	41 (93%)
≥2	12 (14%)	3 (6.8%)
*EGFR* mutant type		
E19 only	39 (45%)	16 (36%)
E21 only	38 (44%)	25 (57%)
Others	9 (10%)	3 (6.8%)
*TP53* mutant		
Yes	44 (51%)	11 (25%)
No	24 (28%)	8 (18%)
Undetected or Unknown	18 (21%)	25 (57%)

Abbreviation: *EGFR*, epidermal growth factor receptor; TKI, tyrosine kinase inhibitor; CR, complete response; PR, partial response; SD, stable disease; PD, progression disease.

## Data Availability

The datasets used in this study are all publicly available.

## Supplementary Materials

The following supporting information can be downloaded at: https://www.mdpi.com/article/10.3390/curroncol30090616/s1, Supplementary Figure S1. Flowchart of detailed inclusion and exclusion criteria. Supplementary Figure S2. Distribution of *EGFR* mutation abundance and Kaplan–Meier survival curves for duration of drug application (DDA). Distribution of *EGFR* mutation abundance in LUAD patients in different clinical groups: (A) *EGFR* mutation number and (C) *EGFR* mutation type. Kaplan–Meier survival curves for the duration of drug application (DDA) in different clinical groups, (B) *EGFR* mutation number, and (D) *EGFR* mutation type. *EGFR*, epidermal growth factor receptor; NS, not significant. Supplementary Figure S3. Kaplan–Meier survival curves for the duration of drug application (DDA) for different *EGFR* (A) and *TP53* (B) mutation abundances after PSM analysis. *EGFR*, epidermal growth factor receptor; PSM, propensity score matching. Supplementary Table S1. Propensity score matching (PSM) analysis results of EGFR mutation abundance. Supplementary Table S2. Propensity score matching (PSM) analysis results of TP53 mutation abundance.
