# Supplementary material for: Concordance of Abundance for Mutational EGFR and Co-Mutational TP53 with Efficacy of EGFR-TKI Treatment in Metastatic Patients with Non-Small-Cell Lung Cancer
Source: Curr Oncol. 2023 Sep 15;30(9):8464–76. doi: 10.3390/curroncol30090616 (PMC10528559; doi:10.3390/curroncol30090616)
Supplement: Supplementary file 1 [file curroncol-30-00616-s001.zip › Supplementary Table 1.docx]

**Supplementary Table S1. Propensity score-matched (PSM) analysis results of EGFR mutation abundance**

| **Characteristics** | **High-EGFR group**  (N = 39) | **Low-EGFR group**  (N = 39) | **Chisq P value** |
| --- | --- | --- | --- |
| **Age (years)** |  |  | 1 |
| <65 | 20 (51%) | 20 (51%) |  |
| ≥65 | 19 (49%) | 19 (49%) |  |
| **Gender** |  |  | 0.82 |
| Male | 20 (51%) | 18 (46%) |  |
| Female | 19 (49%) | 21 (54%) |  |
| **Combine therapy (first-line)** |  |  | 1 |
| No | 34(87%) | 34(87%) |  |
| Yes | 5(13%) | 5(13%) |  |
| **EGFR mutant number** |  |  | 1 |
| 1 | 34 (87%) | 35 (90%) |  |
| ≥2 | 5 (13%) | 4 (10%) |  |
| **EGFR mutant type** |  |  | 0.93 |
| E19 only | 15 (38%) | 16 (41%) |  |
| E21 only | 19 (49%) | 19 (49%) |  |
| Others | 5 (13%) | 4 (10%) |  |
| **TP53 mutant** |  |  | 0.86 |
| Yes | 13 (33%) | 11 (28%) |  |
| No | 11 (28%) | 11 (28%) |  |
| Undetected or Unknown | 15 (38%) | 17 (44%) |  |

**Abbreviation:** EGFR, epidermal growth factor receptor.
