# Supplementary material for: Concordance of Abundance for Mutational EGFR and Co-Mutational TP53 with Efficacy of EGFR-TKI Treatment in Metastatic Patients with Non-Small-Cell Lung Cancer
Source: Curr Oncol. 2023 Sep 15;30(9):8464–76. doi: 10.3390/curroncol30090616 (PMC10528559; doi:10.3390/curroncol30090616)
Supplement: Supplementary file 1 [file curroncol-30-00616-s001.zip › Supplementary Table 2.docx]

**Supplementary Table S2. Propensity score-matched (PSM) analysis results of TP53 mutation abundance**

| **Characteristics** | **High-TP53 group**  (N = 14) | **Low-TP53 group**  (N = 14) | **Chisq P value** |
| --- | --- | --- | --- |
| **Age (years)** |  |  | 1 |
| <65 | 12 (86%) | 11 (79%) |  |
| ≥65 | 2 (14%) | 3 (21%) |  |
| **Gender** |  |  | 1 |
| Male | 6 (43%) | 5 (36%) |  |
| Female | 8 (57%) | 9 (64%) |  |
| **Combine therapy (first-line)** |  |  | 1 |
| No | 12 (85.7%) | 13 (92.9%) |  |
| Yes | 2 (14.3%) | 1 (7.1%) |  |
| **EGFR mutant number** |  |  | 1 |
| 1 | 13 (92.9%) | 13 (92.9%) |  |
| ≥2 | 1 (7.1%) | 1 (7.1%) |  |
| **EGFR mutant type** |  |  | 1 |
| E19 only | 10 (71.4%) | 10 (71.4%) |  |
| E21 only | 3 (21.4%) | 3 (21.4%) |  |
| Others | 1 (7.2%) | 1 (7.2%) |  |
| **EGFR mutant** |  |  | 1 |
| High | 13 (92.9%) | 13 (92.9%) |  |
| Low | 1 (7.1%) | 1 (7.1%) |  |

**Abbreviation:** EGFR, epidermal growth factor receptor.
