# Supplementary figures and images for: Concordance of Abundance for Mutational EGFR and Co-Mutational TP53 with Efficacy of EGFR-TKI Treatment in Metastatic Patients with Non-Small-Cell Lung Cancer
Source: Curr Oncol. 2023 Sep 15;30(9):8464–76. doi: 10.3390/curroncol30090616 (PMC10528559; doi:10.3390/curroncol30090616)

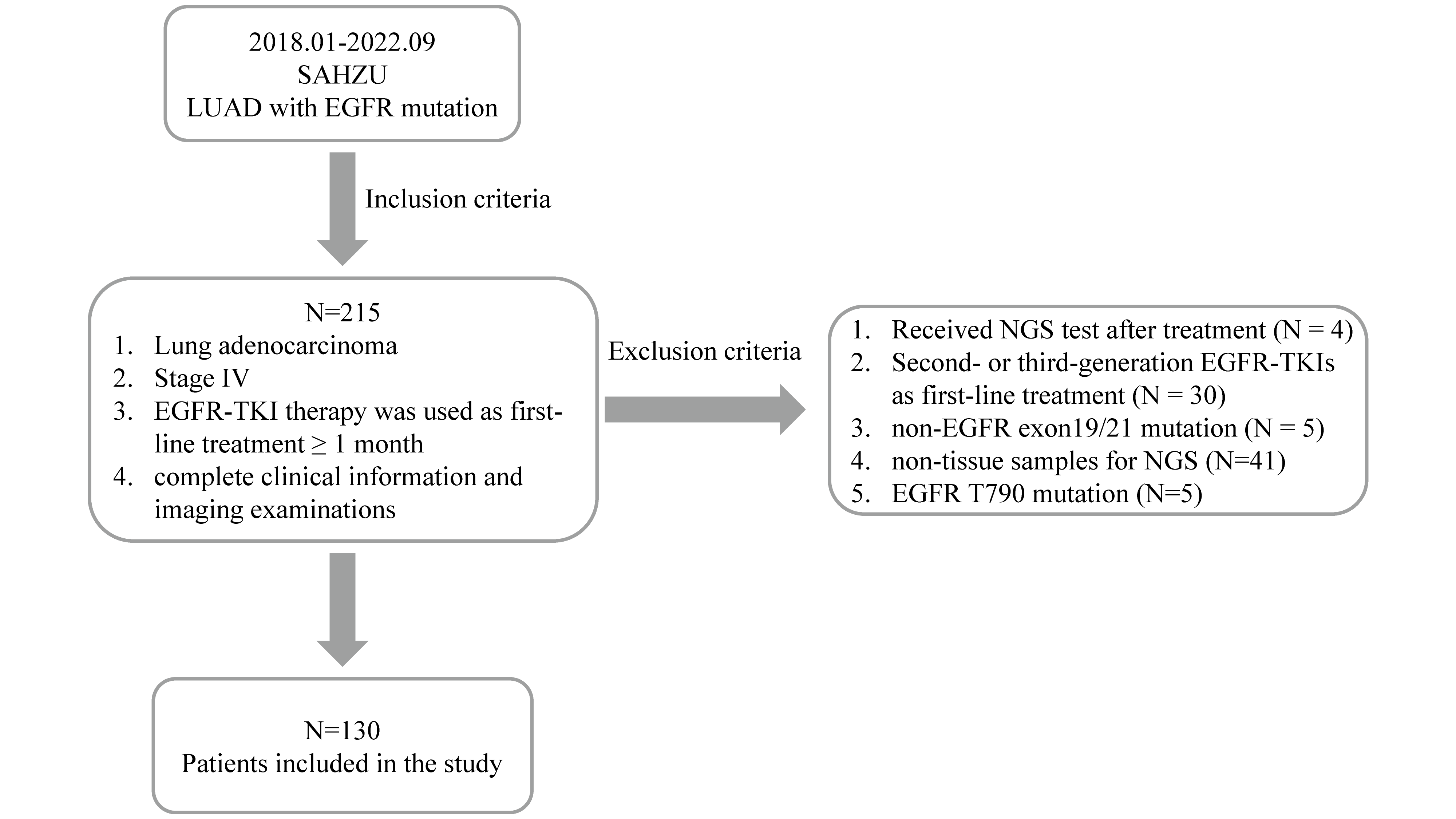

Supplement: Supplementary file 1 [file curroncol-30-00616-s001.zip › Supplementary Figure 1.tif]

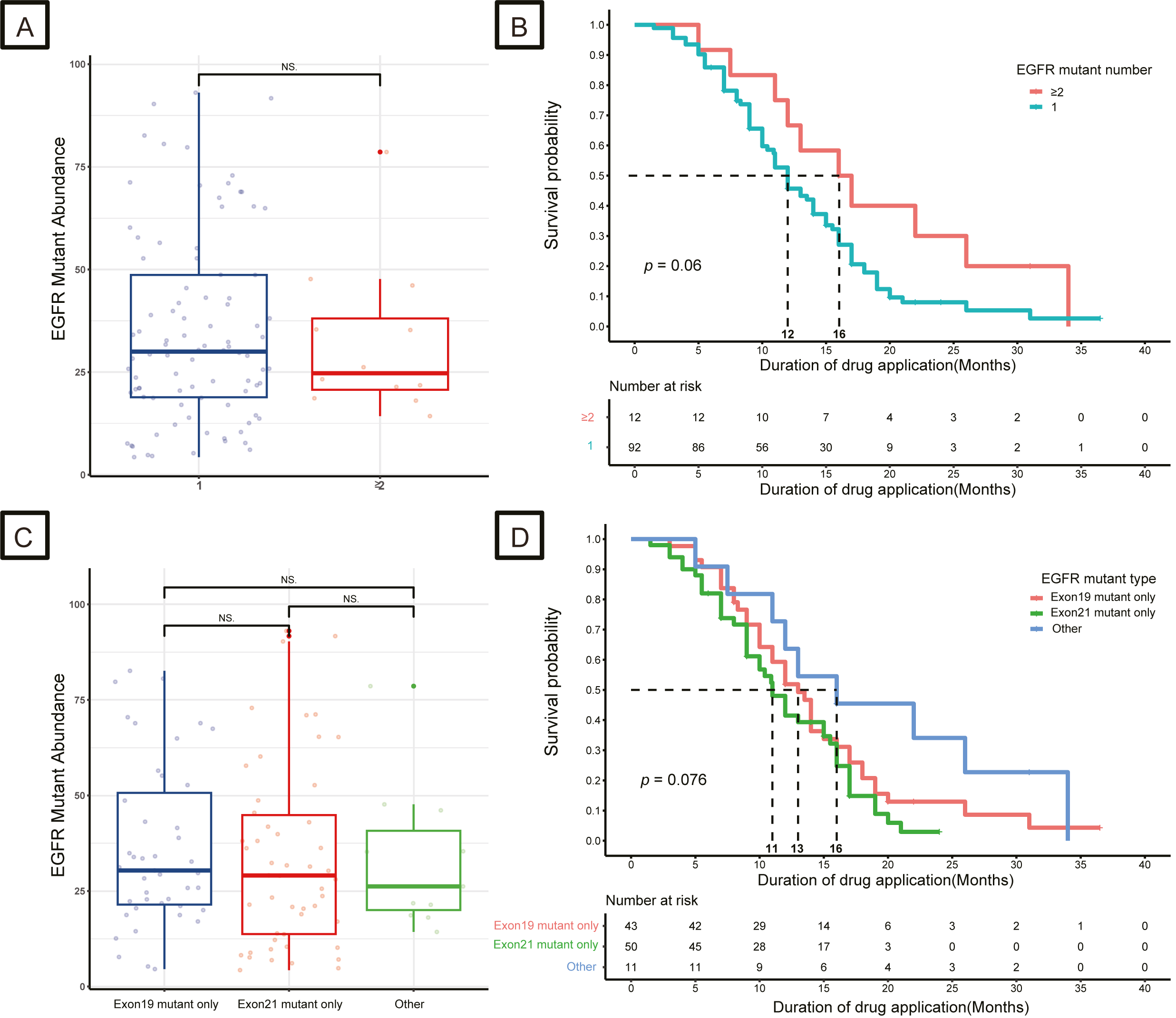

Supplement: Supplementary file 1 [file curroncol-30-00616-s001.zip › Supplementary Figure 2.tif]

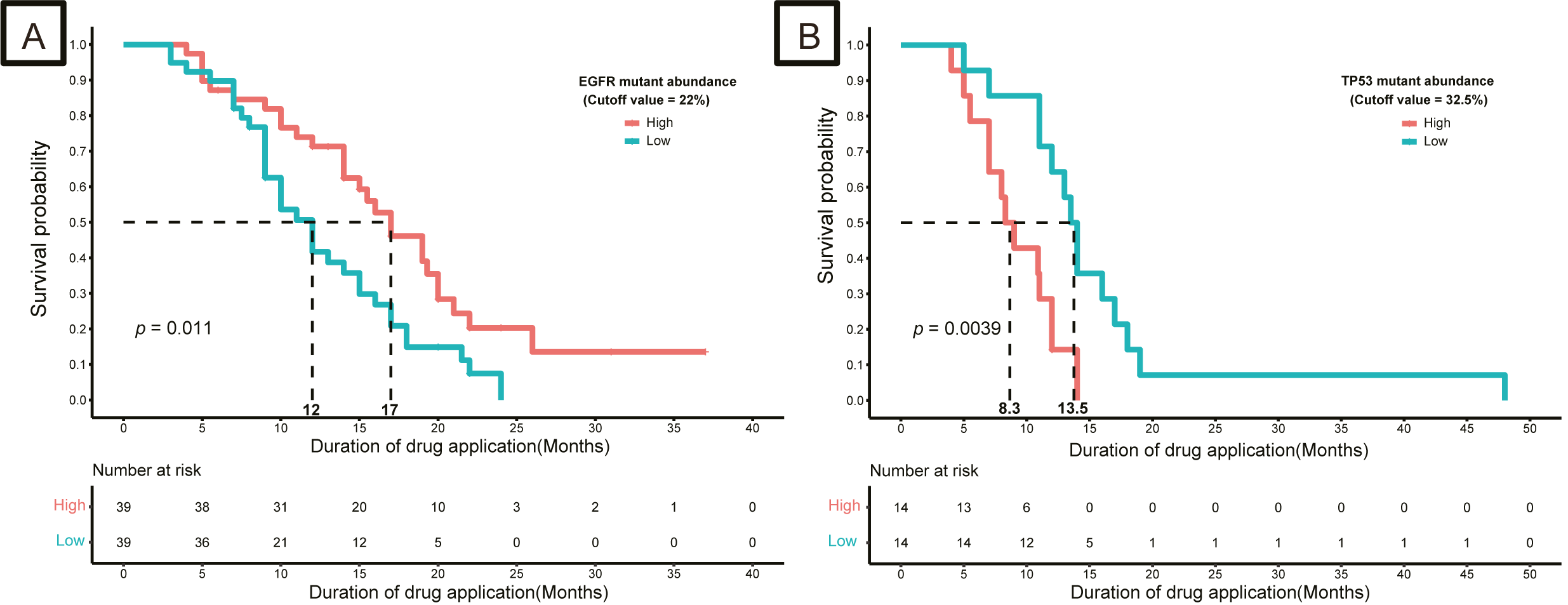

Supplement: Supplementary file 1 [file curroncol-30-00616-s001.zip › Supplementary Figure 3.tif]
